# Association of Hormone Replacement Therapy with Inflammatory Bowel Disease Risk in Women with Menopausal Disorders: A Population-Based Retrospective Cohort Study

**DOI:** 10.3390/healthcare13050578

**Published:** 2025-03-06

**Authors:** Yuan-Tsung Tseng, I-I Chen, Chun-Hsiang Wang

**Affiliations:** 1 Department of Public Health, College of Medicine, National Cheng Kung University, Tainan City 701, Taiwan; 2a0074@tmh.org.tw; 2 Department of Medical Research, Tainan Municipal Hospital (Managed by Show Chwan Medical Care Corporation), Tainan City 701, Taiwan; 3 Department of Hepatogastroenterology, Tainan Municipal Hospital (Managed by Show Chwan Medical Care Corporation), No. 670, Chongde Road, East District, Tainan City 701, Taiwan; 2lm466@tmh.org.tw

**Keywords:** hormone replacement therapy, inflammatory bowel disease, postmenopausal women, ulcerative colitis, Crohn’s disease

## Abstract

**Background**: The long-term effects of hormone replacement therapy (HRT) on inflammatory bowel disease (IBD) remain unclear, necessitating further investigations of the association between HRT and the development of ulcerative colitis and Crohn’s disease in postmenopausal women. **Methods**: This retrospective cohort study utilized Taiwan’s National Health Insurance claims (2001–2018) to identify postmenopausal women aged ≥ 50 years with HRT use. A one-year washout period was applied before the index date to ensure new HRT users. To address the immortal time bias, follow-up for HRT users began at HRT initiation. The non-HRT group was selected by 1:1 propensity score matching. Cox proportional hazards models with adjustments for comorbidities and medications were used to estimate hazard ratios. **Results**: A total of 10,126 postmenopausal women (5063 per group) were included. During a mean follow-up of 11.1 years, the incidence rates of ulcerative colitis were 0.14 and 0.11 per 1000 person-years in the HRT and non-HRT groups, respectively. The adjusted hazard ratios were 1.33 (95% CI, 0.46–3.83; *p* = 0.600) for ulcerative colitis and 0.72 (95% CI, 0.45–1.16; *p* = 0.177) for Crohn’s disease. **Conclusions**: This longitudinal study suggests that HRT use is not significantly associated with the risk of IBD among postmenopausal women. These findings indicate that IBD risk may not need to be a primary concern when considering HRT in this population.

## 1. Introduction

Inflammatory bowel disease (IBD) encompasses Crohn’s disease (CD) and ulcerative colitis (UC), both of which are chronic inflammatory disorders that affect the gastrointestinal tract and are experiencing a rising global incidence. Although genetic factors are acknowledged as pivotal in IBD pathogenesis, it is increasingly evident that environmental influences also hold significant sway over the development of IBD [1,2].

A few epidemiological studies have indicated a correlation between exogenous hormone use and susceptibility to CD and UC. Hormone replacement therapy (HRT) has been identified as a potential contributor. Given that the occurrence of CD and UC demonstrates sex-based disparities, it has been postulated that environmental factors, such as hormone exposure, might partially contribute to these differences [3,4]. The potential impact of exogenous hormone use on IBD pathogenesis has been a subject of conjecture underpinned by diverse mechanisms. One prevailing hypothesis posits that oral estrogen, an ingredient in contraceptives and HRT, can influence intestinal permeability, which is a pivotal factor in the pathogenesis of IBD [5,6]. Furthermore, hormones can act as modulators of the immune response. The use of exogenous hormones may induce alterations in endogenous hormone levels, consequently influencing the emergence of Th1- and Th2-mediated inflammatory disorders [7,8].

Some studies suggest that postmenopausal hormone replacement therapy can protect IBD patients against disease activity. However, the relationship between hormones and IBD is complex, and may have distinct effects on UC and CD. For instance, hormone therapy increases the risk of UC but not CD, suggesting varying mechanisms underlying these diseases [1,9]. Moreover, women with IBD face specific challenges related to reproductive health, including menstrual irregularities, fertility concerns, and contraception. Sex hormone fluctuations during the menstrual cycle and hormonal therapies may also affect disease activity in female IBD patients [4]. Furthermore, postmenopausal hormone use is associated with an increased risk of diverticulitis [10]. Hormones play a complex role in IBD etiology and management, particularly in sex-specific care [4,11]. There are ongoing controversies regarding the enduring ramifications of HRT and IBD. Therefore, this study aimed to longitudinally explore the effects of hormone replacement therapy on IBD by using a new user design.

## 2. Materials and Methods

### 2.1. Data Sources

This study utilized medical claims data from Taiwan’s National Health Insurance (NHI) program, managed by the National Health Insurance Administration. The NHI program provides coverage to over 99% of Taiwan’s population. The 2-million-subject sampled dataset from the National Health Insurance Research Database (NHIRD) is a standard cohort randomly selected by the Ministry of Health and Welfare of Taiwan. This cohort is designed to be representative of the entire insured population and has been widely used in epidemiological studies. The dataset included detailed information on healthcare services, procedures, and prescribed medications documented from 1 January 2000 to 31 December 2018. Medical claims employ the International Classification of Disease, Ninth Revision, Clinical Modification (ICD-9-CM) (before 2016) and Tenth Revision, Clinical Modification (ICD-10-CM) (2016), and therefore coding systems to document diagnoses and establish a solid foundation for the analysis [12,13].

Owing to the encrypted features of the medical claims used in this study, it was not feasible to ascertain the identities of individual patients, thereby eliminating the need to obtain informed consent. The study protocol was approved by the Institutional Review Board (IRB) of the Show Chwan Memorial Hospital on 30 August 2019 (IRB-No: 1080703), ensuring compliance with ethical standards.

### 2.2. Study Design and Definition of Study Cohort

A cohort study design was used to investigate the association between HRT and IBD in postmenopausal women. Specifically, postmenopausal women were identified based on ICD-9-CM codes 627.1–627.9 or ICD-10-CM code N95, with at least three recorded occurrences of these diagnoses during the study period. The HRT group comprised individuals who received prescriptions for HRT, including estrogens (e.g., conjugated estrogen and estradiol) and progestins (e.g., progesterone, norepinephrine, cyproterone, ethisterone, medroxyprogesterone, and norgestrel). Postmenopausal women over 50 years of age who were diagnosed with the specified disease codes at least three times during the study period were identified. To examine potential duration-dependent associations, the HRT group was further stratified into two subgroups based on cumulative exposure: short-term users (<1 year of cumulative use) and long-term users (≥1 year of cumulative use). The cumulative duration was calculated from the total number of prescribed days of HRT during the study period.

**Figure 1:**
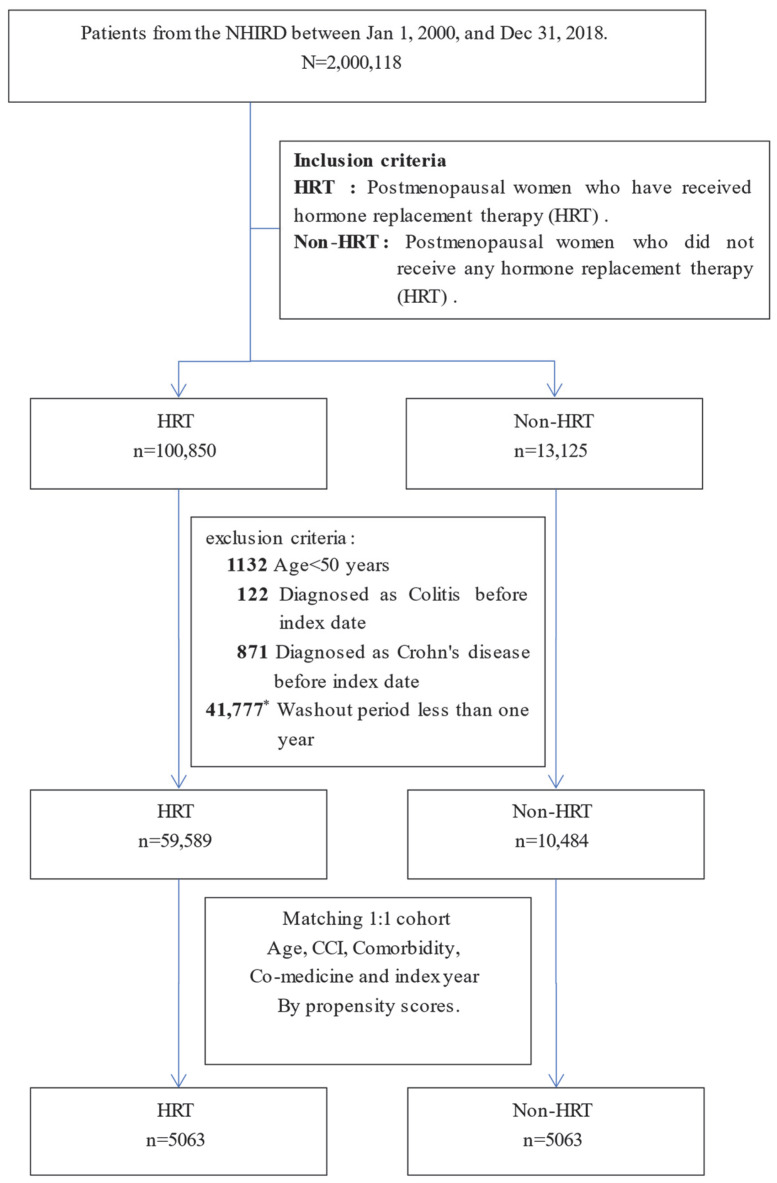
Flowchart of postmenopausal patient selection for HRT and non-HRT cohorts. * The overall dataset spans 2000–2018, but the 2000 dataset was used as a washout period to exclude prior cases, and the actual patient selection period was 2001–2017. Patients were followed until 2018 to ensure at least one year of follow-up. Abbreviations: HRT, hormone replacement therapy; DM, diabetes mellitus; CCI: Charlson Comorbidity Index; index year, exact year of diagnosis.

The non-HRT group consisted of postmenopausal women with no recorded use of HRT during the study period. The women were identified using the same inclusion criteria as those in the HRT group. Participants with prior diagnoses of IBD, CD, or UC before the index date or those who failed to complete the one-year washout period were excluded from the study. To ensure balanced baseline characteristics between the two groups, we accounted for patients’ medical history, including age, comorbidities, and concomitant medications. These variables were incorporated into the propensity score model to match HRT users with comparable non-HRT users, with both groups starting follow-up in the same index year (Figure 1).

### 2.3. Definition of Study Periods and Bias Mitigation Methods

The dataset used for this study spans from 1 January 2000 to 31 December 2018. However, to ensure the inclusion of newly diagnosed patients, a one-year washout period (2000) was implemented, excluding individuals with prior diagnoses before 1 January 2001. Therefore, the actual patient selection period was from 1 January 2001 to 31 December 2017.

The index date for HRT users was defined as the date of the first HRT prescription following this one-year washout period, ensuring that all individuals were new users. The non-HRT group was matched based on the same index year to maintain temporal consistency between the groups.

To mitigate immortal time bias, follow-up for HRT users began precisely at the time of HRT initiation, preventing pre-treatment periods from being included. A minimum one-year follow-up period after the index date was required to account for the induction time necessary for HRT to influence IBD risk. Therefore, patients were followed until 31 December 2018, ensuring sufficient observation time.

It is important to note that while the overall dataset spans 2000–2018, patients diagnosed in 2018 were not included in the cohort, as they would not have met the minimum one-year follow-up requirement. The 2018 data were used solely to extend the follow-up period for individuals diagnosed earlier.

### 2.4. Main Outcome and Covariate Measurements

We investigated the primary outcomes of our study by focusing on two distinct conditions: ulcerative colitis (UC; ICD-9 code 556.x; ICD-10 code K51) and Crohn’s disease (CD; ICD-9 code 555.x; ICD-10 code K50). A confirmed diagnosis of each condition was defined as having at least three recorded diagnoses in the National Health Insurance Research Database (NHIRD) claims data to enhance diagnostic accuracy. According to previous population-based research in Taiwan, lower gastrointestinal (GI) endoscopy, upper GI endoscopy, and abdominal ultrasonography are commonly used to exclude organic gastrointestinal diseases before diagnosing IBS [14].

To mitigate the influence of potential confounders, we carefully identified and accounted for the following comorbidities: hypertension (ICD-9 codes 401–405; ICD-10 codes I10–I15), hyperlipidemia (ICD-9 code 272; ICD-10 code E78), diabetes mellitus (DM; ICD-9 code 250; ICD-10 codes E10.0, E10.1, E10.9, E11.0, E11.1, and E11.9), and chronic kidney disease (CKD; ICD-9 code 585; ICD-10 code N18). The Charlson Comorbidity Index (CCI) was among the key health condition indices evaluated.

### 2.5. Statistical Method

Differences in baseline characteristics, including prescriptions and medical records, as continuous variables were assessed using *t*-tests. The chi-square test was used to compare categorical variables. To minimize potential selection bias, propensity score matching (PSM) was used to balance the proportions of comorbidities between the HRT and non-HRT cohorts.

A 1:1 propensity score matching without replacement was performed to ensure strict matching and minimize selection bias. The regression model was tailored to incorporate the treatment, and all covariates were identified for matching. No weighting or trimming method was applied to this process [15]. For propensity score matching (PSM), we executed both multivariate logistic regression analysis and nearest-neighbor matching, setting the tolerance level to zero, using the “MatchIt” package in R (version 4.3.4).

Bootstrapping techniques were implemented to adjust the hazard ratios (HRs) and their respective 95% confidence intervals (CIs) to bolster the model’s robustness and stability. Cox proportional hazards regression models were then used to assess the risk associated with the target outcome in HRT users compared to those not using HRT, estimating HRs and 95% CIs [16]. The Kaplan–Meier method was used to estimate the outcomes of different study cohorts, and differences in survival curves were assessed using the log-rank test. For the sensitivity analysis, we applied both the Kaplan–Meier method and Cox proportional hazards regression models. Data management and processing were carried out using SPSS (version 21.0; SPSS Inc., Chicago, IL, USA) and R (version 3.4.3; R Core Team, Vienna, Austria).

## 3. Results

### 3.1. Patient Characteristics and Balanced Groups

Initially, 113,975 postmenopausal patients diagnosed between 1 January 2001 and 31 December 2017 were identified. The study dataset spans from 1 January 2000 to 31 December 2018, but the 2000 dataset was used exclusively as a washout period, ensuring that patients had no prior history of the disease before entering the cohort. Patients diagnosed after 31 December 2017 were excluded to guarantee a minimum of one year of follow-up. The final study population was followed until 31 December 2018 (Figure 1).

Ultimately, a well-defined cohort of 10,126 postmenopausal women met the inclusion criteria (Figure 1). The comprehensive demographic profiles of the study and matched cohorts (maintained at a 1:1 patient ratio) are detailed in Table 1. The mean age of both groups was 62.5 ± 8.7 years, with a central tendency observed at 61 years. The mean follow-up duration was meticulously calculated, resulting in 11.1 ± 5.07 years (median: 11.3) for the HRT group and 11.0 ± 5.08 years (median: 11.2) for the non-HRT group.

Demographic characteristics (such as age and Charlson Comorbidity Index) and comorbid conditions (including hypertensive cardiovascular disease, hyperlipidemia, diabetes mellitus, and chronic kidney disease) were uniformly distributed across both cohorts. Concurrent medications were evenly matched across both groups, including aspirin, statins, angiotensin-converting enzyme inhibitors (ACEIs), β-blockers, spironolactone, glucocorticoids, and selective serotonin reuptake inhibitors (SSRIs).

### 3.2. Comparison of Incidence Rates of UC and CD

Table 2 presents the incidence rates of UC and CD in the HRT and non-HRT groups. The incidence rates, expressed per 1000 person-years, are presented alongside their corresponding 95% confidence intervals (CIs).

**Figure 2:**
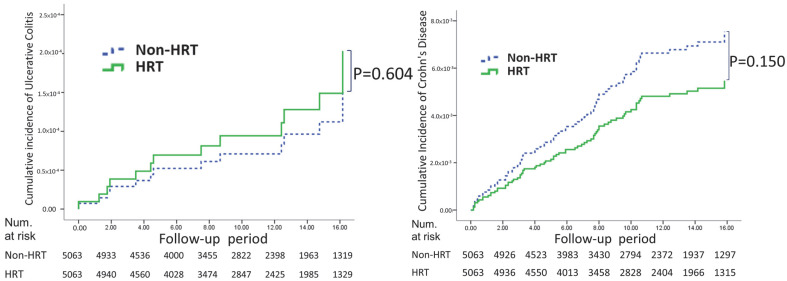
Comparative cumulative incidence of ulcerative colitis and Crohn’s disease in HRT and non-HRT cohorts over the follow-up period.

For UC, the HRT cohort exhibited an incidence rate of 0.14 (95% CI 0.07–0.28), while the non-HRT cohort had a rate of 0.11 (95% CI 0.04–0.24). In the case of CD, the incidence rate was 0.54 (95% CI 0.37–0.76) in the HRT cohort and 0.75 (95% CI 0.56–1.02) in the non-HRT cohort. In Figure 2, the log-rank tests indicate no significant difference in cumulative risk between the HRT and non-HRT groups for UC (*p* = 0.604) and Crohn’s disease (*p* = 0.150).

### 3.3. UC and CD Incidence by HRT Duration

We examined the incidence of UC and CD among the HRT and non-HRT cohorts stratified according to the duration of HRT use (Table 3). We report the hazard ratios (HRs) with their corresponding 95% confidence intervals (CIs), using the non-HRT cohort as the reference group.

For UC, the HR for the HRT cohort with any duration of use was 1.33 (95% CI 0.46–3.83), indicating no statistically significant deviation in UC risk compared to the non-HRT cohort. Stratification by HRT duration yielded HRs of 1.53 (95% CI 0.49–4.76) for HRT use of less than 1 year and 0.95 (95% CI 0.19–4.73) for HRT use of 1 year or more. Similar patterns were observed for CD, with HRs of 0.72 (95% CI 0.45–1.16) for any HRT use, 0.78 (95% CI 0.46–1.32) for HRT use of less than 1 year, and 0.62 (95% CI 0.30–1.27) for HRT use of 1 year or more.

**Figure 3:**
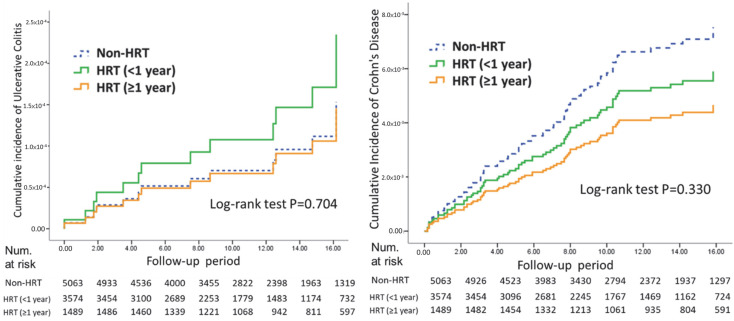
Cumulative incidence of ulcerative colitis and Crohn’s disease by HRT exposure duration compared to non-HRT cohorts over the follow-up period.

While the cumulative incidence risks for UC were comparable between the HRT and non-HRT groups (*p* = 0.704), and although the cumulative risk of CD appeared lower in the HRT group, none of these differences reached statistical significance (log-rank test, *p* = 0.330) (Figure 3).

### 3.4. Associations with UC and CD

We evaluated the associations between various factors and the incidence of UC and CD through comprehensive analysis using a multiple Cox regression model and propensity score matching. Table 4 presents the detailed results of these associations, along with the respective *p*-values, indicating the statistical significance of the contribution of each factor to the diseases.

Age displayed a minimal association with UC risk, with a hazard ratio (HR) of 1.02 (95% CI 0.95–1.09, *p* = 0.621). For CD, age exhibited a slightly significant HR of 1.04 (95% CI 1.01–1.07, *p* = 0.010). The CCI, hypertension, hyperlipidemia, DM, CKD, and medication regimens including aspirin, statins, ACE inhibitors, beta-blockers, spironolactone, and SSRIs showed no significant association with the incidence of UC or CD.

## 4. Discussion

Our large-scale population-based cohort study found no significant association between HRT use and IBD risk in postmenopausal women. This finding contributes evidence to the ongoing debate about the relationship between exogenous hormones and inflammatory bowel disease

### 4.1. Comparison with Existing Literature

Our study found no significant association between hormone replacement therapy (HRT) use and IBD risk in postmenopausal women. This lack of association contributes important evidence to the ongoing debate regarding the relationship between exogenous hormones and inflammatory bowel disease (IBD). Previous studies have reported conflicting results, with some suggesting increased IBD risk among HRT users [17,18,19], while others indicate potential protective effects [9]; our results indicate that such benefits may not extend to disease incidence. There is a disparity between our findings and those reporting protective effects [20]. Our results align with neither perspective, suggesting a more nuanced relationship between hormone therapy and IBD risk.

Several studies have reported elevated IBD risk associated with HRT use, particularly in specific patient subgroups [17,18]. The mechanisms proposed include potential enhancement of pro-inflammatory pathways and alterations in intestinal barrier function [19]. However, our findings do not support these associations, even when analyzing different durations of HRT exposure. This disparity might be attributed to differences in study populations, HRT formulations, or methodological approaches. Our lack of association also contrasts with studies suggesting protective effects, which primarily focused on disease activity rather than initial occurrence. Our findings suggest that while HRT influences immune responses, it does not necessarily increase IBD incidence.

### 4.2. Biological Mechanisms and Study Implications

While our study does not support a protective role of HRT in IBD prevention, the complex biological interactions between sex hormones and intestinal inflammation merit discussion. Previous research has described mechanisms through which estrogen might influence inflammatory processes, including nuclear factor-κB (NF-κB) inhibition and cytokine modulation [21,22]. Furthermore, estrogen receptors, particularly ERβ, have been implicated in maintaining intestinal barrier integrity and modulating immune cell function in the gut mucosa. Research has demonstrated that estradiol decreases colonic permeability through estrogen receptor β-mediated up-regulation of occludin and junctional adhesion molecule-A in epithelial cells [23,24].

It is important to note that our epidemiological study design, while suitable for examining population-level associations, was not designed to observe or verify these molecular mechanisms. The complex nature of IBD pathogenesis involves multiple cellular and molecular pathways that may not be fully captured in observational studies. Additionally, potential effects of HRT on these molecular pathways might be influenced by various factors such as the timing of hormone exposure, individual genetic variations, and environmental influences that were beyond the scope of our current investigation. Therefore, our lack of association should not be interpreted as evidence against these molecular mechanisms, but rather as a limitation in our ability to detect their clinical manifestations in our study setting.

### 4.3. Clinical Implications

From a clinical perspective, our findings suggest that IBD risk should not be a primary concern when prescribing HRT to postmenopausal women. While previous research has suggested potential sex hormone influences on IBD progression [1,4], our study indicates that HRT use does not substantially alter the risk of developing IBD. This information is crucial for clinicians when counseling postmenopausal women considering HRT for symptom relief, as concerns regarding IBD risk may not need to influence treatment decisions.

Additionally, given the inconsistencies in the literature, it remains essential for healthcare providers to adopt a personalized approach to HRT prescription, considering other risk factors such as cardiovascular disease, osteoporosis, and individual patient history [25,26]. Future studies should aim to examine whether specific HRT formulations or administration routes (e.g., oral vs. transdermal) differentially affect inflammatory processes in the gut.

### 4.4. Limitations

Our study has several important limitations that warrant consideration when interpreting our findings. First, the NHI database did not include data on critical lifestyle and dietary factors that play vital roles in IBD etiology, including Western diet consumption, processed food intake, and fiber consumption, which have been implicated in IBD pathogenesis. Additionally, determining the socioeconomic status of the participants posed challenges owing to the lack of comprehensive NHI data. Second, the database used did not provide specific information on laboratory test results, which could have provided valuable insights into the disease severity and biomarker profiles. Third, potential misclassification of ICD codes may have occurred, leading to the misdiagnosis of comorbidities in our study. Fourth, family history—an important risk factor for IBD—was not available in the NHI database, limiting our ability to account for genetic predisposition in our analyses. Fifth, the generalizability of our findings may be influenced by racial and geographical factors. IBD prevalence, genetic susceptibility, and environmental exposures vary across different populations and regions, which may impact the applicability of our results. Furthermore, the accuracy of medication compliance for HRT could not be precisely measured using NHI data, which potentially affected our estimation of prescription usage. Finally, given the limited information available on over-the-counter HRT use, our study may have underestimated the extent of exposure. These limitations, including the lack of lifestyle factors and potential ICD code misclassifications, should be carefully considered when interpreting our findings.

## 5. Conclusions

This longitudinal cohort study found no significant association between HRT and IBD risk. Despite the theoretical mechanisms linking hormonal factors to IBD pathogenesis, our findings suggest that HRT use does not substantially influence IBD risk in postmenopausal women. Therefore, when considering HRT for postmenopausal women, concerns regarding IBD risk may not require significant emphasis.

## Figures and Tables

**Table 1:** Baseline characteristics of postmenopausal women in HRT and non-HRT cohorts after propensity score matching ^†^.

Characteristic	Non-HRT (n = 5063)	HRT (n = 5063)
Demographics		
Age, years ^‡^	62.5 ± 8.7	62.5 ± 8.7
CCI ^‡^	1.74 ± 2.0	1.74 ± 2.0
Comorbidities, n (%)		
Hypertension	1818 (35.9)	1818 (35.9)
Hyperlipidemia	1957 (38.7)	1957 (38.7)
DM	887 (17.5)	887 (17.5)
CKD	100 (2.0)	100 (2.0)
Medications, n (%)		
Aspirin	1509 (29.8)	1509 (29.8)
Statins	1531 (30.2)	1531 (30.2)
ACEIs	1142 (22.6)	1142 (22.6)
β-blockers	2915 (57.6)	2915 (57.6)
Spironolactone	176 (3.5)	176 (3.5)
Glucocorticoids	4494 (88.8)	4494 (88.8)
SSRIs	674 (13.3)	674 (13.3)

Abbreviations: HRT, hormone replacement therapy; CCI, Charlson Comorbidity Index; DM, diabetes mellitus; CKD, chronic kidney disease; ACEIs, angiotensin-converting enzyme inhibitors; SSRIs, selective serotonin reuptake inhibitors. ^†^ Propensity score matching (1:1 nearest-neighbor matching without replacement) was used to balance baseline characteristics between the HRT and non-HRT groups. ^‡^ Values are presented as mean ± standard deviation. Categorical variables are presented as numbers (percentages).

**Table 2:** Incidence rates of ulcerative colitis and Crohn’s disease among postmenopausal women in HRT and non-HRT cohorts.

End Point	HRT	IR ^†^	(95% CI)	Non-HRT	IR ^†^	(95% CI)
Ulcerative colitis	8	0.14	(0.07–0.28)	6	0.11	(0.04–0.24)
Crohn’s disease	30	0.54	(0.37–0.76)	42	0.75	(0.56–1.02)

^†^ Incidence rates were calculated per 1000 person-years, and confidence intervals (CIs) were estimated using the Poisson distribution. Abbreviations: HRT, hormone replacement therapy; IR, incidence rate; CI, confidence interval.

**Table 3:** Risk of ulcerative colitis and Crohn’s disease stratified by duration of HRT use ^†^.

HRT Duration	UC		CD	
	HR (95% CI)	*p*-value	HR (95% CI)	*p*-value
Non-HRT (Reference)	1.00	-	1.00	-
Any duration	1.33 (0.46–3.83)	0.600	0.72 (0.45–1.16)	0.177
<1 year	1.53 (0.49–4.76)	0.461	0.78 (0.46–1.32)	0.359
≥1 year	0.95 (0.19–4.73)	0.949	0.62 (0.30–1.27)	0.190

^†^ Hazard ratios (HRs) and 95% confidence intervals (CIs) were estimated using Cox proportional hazards regression models. Abbreviations: HRT, hormone replacement therapy; HR, hazard ratio; CI, confidence interval.

**Table 4:** Adjusted hazard ratios for ulcerative colitis and Crohn’s disease using multivariable Cox regression ^†^.

Factors	UC	*p*	CD	*p*
Age	1.02 (0.95–1.09)	0.621	1.04 (1.01–1.07)	0.010
CCI	1.19 (0.89–1.60)	0.240	1.00 (0.87–1.16)	0.957
Hypertension	3.19 (0.66–15.5)	0.150	2.03 (0.99–4.17)	0.053
Hyperlipidemia	0.43 (0.07–2.80)	0.380	0.87 (0.42–1.82)	0.711
DM	2.90 (0.63–13.4)	0.174	0.63 (0.33–1.23)	0.179
CKD	2.06 (0.33–12.9)	0.440	0.52 (0.12–2.28)	0.385
Aspirin	0.24 (0.05–1.10)	0.066	1.31 (0.73–2.35)	0.366
Statins	0.88 (0.15–5.10)	0.882	0.95 (0.46–1.96)	0.888
ACEIs	0.48 (0.11–2.05)	0.319	0.89 (0.48–1.65)	0.701
β-blockers	1.42 (0.26–7.87)	0.689	1.33 (0.66–2.70)	0.426
Spironolactone	1.41 (0.24–8.16)	0.699	1.86 (0.86–4.03)	0.113
Glucocorticoids	n/a	0.983	2.91 (0.7–12.09)	0.142
SSRIs	2.21 (0.69–7.07)	0.180	0.97 (0.50–1.87)	0.928

^†^ Multivariable Cox proportional hazards regression was used to assess the association between HRT and IBD outcomes, adjusting for potential confounders. Abbreviations: HR, hazard ratio; CI, confidence interval; CCI, Charlson Comorbidity Index; DM, diabetes mellitus; CKD, chronic kidney disease; ACEIs, angiotensin-converting enzyme inhibitors; SSRIs, selective serotonin reuptake inhibitors. n/a: no estimations were obtained using the Cox regression. Boldface indicates statistical significance (*p* < 0.05).

## Data Availability

The primary data for this study were obtained from Taiwan NHI, a population-level dataset managed by the Taiwan Ministry of Health and Welfare. NHI data were fully de-identified and complied with Taiwan’s Personal Data Protection Act to ensure patient privacy. Due to legal and ethical restrictions, direct access to the NHI data was not permitted. Researchers interested in accessing NHI may submit an application to the Ministry of Health and Welfare (https://dep.mohw.gov.tw/dos/cp-5283-63826-113.html accessed on 16 March 2023) with a detailed research proposal and must adhere to strict data use agreements.

## Abbreviations

The following abbreviations are used in this manuscript:

HRT	Hormone replacement therapy
IBD	Inflammatory bowel disease
UC	Ulcerative colitis
CD	Crohn’s disease
NHI	National Health Insurance
ICD	International Classification of Disease
ICD-9-CM	International Classification of Disease, Ninth Revision, Clinical Modification
ICD-10-CM	International Classification of Disease, Tenth Revision, Clinical Modification
CKD	Chronic kidney disease
DM	Diabetes mellitus
CCI	Charlson Comorbidity Index
HR	Hazard ratio
CI	Confidence interval
PSM	Propensity score matching
IRB	Institutional Review Board
SSRI	Selective serotonin reuptake inhibitor
ACEI	Angiotensin-converting enzyme inhibitor
